# Aptamer Probes Labeled with Lanthanide‐Doped Carbon Nanodots Permit Dual‐Modal Fluorescence and Mass Cytometric Imaging

**DOI:** 10.1002/advs.202102812

**Published:** 2021-11-01

**Authors:** Youyi Yu, Xin Wang, Xiaolong Jia, Zijian Feng, Lulu Zhang, Hongxia Li, Jie He, Guangxia Shen, Xianting Ding

**Affiliations:** ^1^ State Key Laboratory of Oncogenes and Related Genes Institute for Personalized Medicine School of Biomedical Engineering Shanghai Jiao Tong University Shanghai 200030 China; ^2^ Department of Urology Ningbo First Hospital Ningbo Hospital of Zhejiang University Ningbo Zhejiang Province 315700 China

**Keywords:** aptamers, carbon dots, dual‐modal imaging, lanthanide‐doped

## Abstract

High‐dimensional imaging mass cytometry (IMC) enables simultaneous quantification of over 35 biomarkers on one tissue section. However, its limited resolution and ultralow acquisition speed remain major issues for general clinical application. Meanwhile, conventional immunofluorescence microscopy (IFM) allows sub‐micrometer resolution and rapid identification of the region of interest (ROI), but only operates with low multiplicity. Herein, a series of lanthanide‐doped blue‐, green‐, and red‐fluorescent carbon nanodots (namely, B‐Cdots(Ln_1_), G‐Cdots(Ln_2_), and R‐Cdots(Ln_3_)) as fluorescence and mass dual‐modal tags are developed. Coupled with aptamers, B‐Cdots(^159^Tb)‐A10‐3.2, G‐Cdots(^165^Ho)‐AS1411, and R‐Cdots(^169^Tm)‐SYL3C dual‐functional aptamer probes, which are then multiplexed with commercially available Maxpar metal‐tagged antibodies for analyzing clinical formalin‐fixed, paraffin‐embedded (FFPE) prostatic adenocarcinoma (PaC) tissue, are further synthesized. The rapid identification of ROI with IFM using fluorescence signals and subsequent multiplexed detection of in situ ROI with IMC using the same tissue section is demonstrated. Dual‐modal probes save up to 90% IMC blind scanning time for a standard 3.5 mm × 3.5 mm overall image. Meanwhile, the IFM provides refined details and topological spatial distributions for the functional proteins at optical resolution, which compensates for the low resolution of the IMC imaging.

## Graphical abstract

The MC‐Cdots(Ln)‐aptamers serve as dual‐modal probes that realize the rapid identification of region of interest (ROI) with immunofluorescence microscopy using fluorescence signal, and then multiplexed detection of in situ ROI with imaging mass cytometry on the same tissue section.

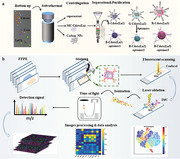

## 1 Introduction

Imaging mass cytometry (IMC) has rapidly evolved into a mainstream technique for the high‐dimensional analysis of tissue sections. IMC currently measures up to over 35 biological parameters simultaneously with subcellular spatial resolution.^[^ ^1^ ^]^ Tissue sections or cultured cells immobilized on glass slides are stained with panels of antibodies mainly labeled by commercially available lanthanide isotopes‐chelating polymer (MCP(Ln)) and inserted into an ablation chamber where the tissue is scanned spot‐by‐spot along a scan line, while sequential scan lines ultimately yield an intensity map of all target proteins across the ROI. The speed of laser ablation is typically 200 pixels s^−1^, which requires ≈0.35 h to produce an area of 0.5 mm × 0.5 mm imaging at 1‐µm resolution.^[^ ^2^ ^]^

This ultralow speed sets a practical limit to the coverage of a tissue section that can be scanned, and it is prohibitive for general clinical application. Therefore, immunocytochemistry (ICC) and immunohistochemistry (IHC) are often required to first locate the ROI based on several identity biomarkers, and then metal‐labeled antibodies are stained on another serial section for IMC multiplexed analysis.^[^ ^1^, ^3^ ^]^ However, the differences in serial sections inevitably result in inaccurate spatial localization. Here, we demonstrate a solution by developing fluorescence and mass dual‐modal probes, which enables rapid scanning using IFM (h/500 mm^2^) to locate the ROI, followed by IMC detection for high‐dimensional proteomic information in situ, thus withdrawing the necessity for rare sample serial sections, minimizing the wasted time of blind sweep during the IMC acquisition, and improving the spatial accuracy of analysis. Meanwhile, IFM enables the visualization of the functional proteins with detailed spatial distribution at nanometer resolution.^[^ ^4^, ^5^, ^6^ ^]^ Overall, IFM and IMC are highly complementary imaging technologies, and a dual‐modal probe that functions in both IFM and IMC will troubleshoot issues encountered in either technology used alone. Here, we describe a new technique by designing dual‐functional fluorescence and mass probes that offer multicolor‐emitting fluorescence signals and multimass signals.

Fluorescent carbon dots (Cdots, 2–10 nm) have been reported as inexpensive fluorescent imaging probes,^[^ ^7^, ^8^, ^9^, ^10^, ^11^ ^]^ and they are reported to have desired biocompatibility, low toxicity, and tunable emission. In particular, as there are abundant hydroxyl, carboxylic, and aldehyde groups on the surface of Cdots, the conjugation of small oligonucleotide ligands (aptamers), ^[^ ^12^ ^]^ peptides, or antibodies is readily versatile, making Cdots ideal materials for fluorescent imaging, drug delivery,^[^ ^13^ ^]^ and cancer therapy.^[^ ^14^, ^15^, ^16^, ^17^, ^18^ ^]^ The modifiable carbon‐based materials broaden the types of the molecular probes and affinity reagents, compared with the commercial MCPs(Ln) mass tags, which are only coupled with sulfhydryl groups located in the hinge region between two heavy chains of the IgG antibody.

We designed and synthesized lanthanide‐doped multicolor fluorescent carbon nanodots (MC‐Cdots(Ln)) with fluorescence and mass dual signals. The lanthanide ions were first coordinated with oxygen atoms of citric acid (CA) and urea in dimethyl formamide (DMF) solvent for an hour. The mixture was immediately carbonized to form stable MC‐Cdots(Ln). The MC‐Cdots(Ln) exhibited excitation‐dependent photoluminescence (PL) spectra similar to the Cdots.^[^ ^19^ ^]^ We purified the MC‐Cdots(Ln) through size‐exclusion chromatography (SEC) with different elution time, obtaining blue‐, green‐, and red‐emission fluorescent fractions, named as B‐Cdots(Ln), G‐Cdots(Ln), and R‐Cdots(Ln), respectively. The relative PL quantum yields reached up to 42.6%, 8.82%, and 6.92%, respectively. The desired fluorescence properties of the MC‐Cdots(Ln) ensured a bright fluorescence tag for fluorescence imaging. In addition, different lanthanide atoms were doped into Cdots to form a library of dual‐modal tags. Lanthanide‐doped blue‐, green‐, and red‐Cdots (namely, B‐Cdots(Ln_1_), G‐Cdots(Ln_2_), and R‐Cdots(Ln_3_)) were synthesized, with functional carboxyl groups, followed by conjugation with NH_2_‐aptamers through amide reaction to form B‐Cdots(Ln_1_)‐aptamer_1_, G‐Cdots(Ln_2_)‐aptamer_2_, and R‐Cdots(Ln_3_)‐aptamer_3_ dual‐functional probes.

**Scheme 1:**
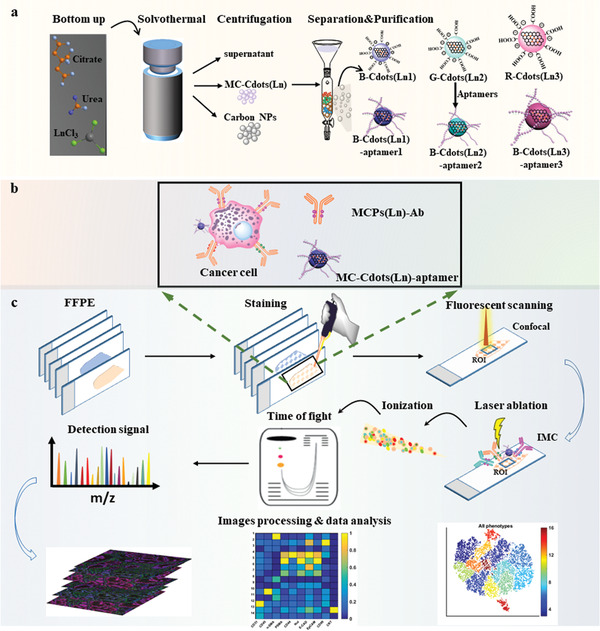
a) Synthesis and purification of lanthanide‐doped multicolor fluorescent carbon nanodots (MC‐Cdots(Ln)) as dual‐functional tags. b) The aptamer probes labeled with MC‐Cdots(Ln) were further multiplexed with commercially available Maxpar metal‐tagged antibodies for labeling cells or tissue sections. c) The MC‐Cdots(Ln)‐aptamers serve as dual‐modal probes that realize the rapid identification of ROI with IFM using fluorescence signal, and then multiplexed detection of in situ ROI with IMC on the same tissue section.

We evaluated the potential of the dual‐modal tags by conjugating A10‐3.2 aptamer against PSMA antigen in PaC cyto‐staining assays. The specificity of B‐Cdots(^165^Ho)‐A10‐3.2 probe was demonstrated in LNCaP cells (PSMA+) by IFM and IMC. In addition, IFM presented a detailed spatial distribution of PSMA, nucleolin (NCL), and anti‐epithelial cell adhesion molecule (EpCAM) in LNCaP. The synthetic B‐Cdots(^159^Tb)‐A10‐3.2, G‐Cdots(^165^Ho)‐AS1411, and R‐Cdots(^169^Tm)‐SYL3C dual‐functional probes were further multiplexed with the commercially available Maxpar metal‐tagged antibodies to validate the dual‐functionality in sections of clinical formalin‐fixed, paraffin‐embedded (FFPE) PaC tissue. We constructed an area of 3.5 mm × 3.5 mm fluorescence images to locate the ROI using tile scanning mode of IFM, and then the marked ROI on the same tissue section was sequentially detected by IMC to obtain the multiplexed proteomic expression using mass signals. The whole process saved about 90% IMC blind scanning time. The use of dual‐modal probes avoids the necessity of serial sections for clinical rare precious samples and largely enhances the overall data acquisition speed, which is particularly essential for general clinical application. The comprehensive workflow is shown in **Scheme** **1**. First, we synthesized and purified a series of lanthanide‐doped multicolor fluorescent carbon nanodots (MC‐Cdots(Ln)) as dual‐functional tags. The purified B‐Cdots(Ln_1_), G‐Cdots(Ln_2_), and R‐Cdots(Ln_3_)) were coupled with NH_2_‐aptamers through amide reaction to form B‐Cdots(Ln_1_)‐aptamer_1_, G‐Cdots(Ln_2_)‐aptamer_2_, and R‐Cdots(Ln_3_)‐aptamer_3_ as dual‐functional probes. Then MC‐Cdots(Ln)‐aptamers probes were further multiplexed with commercially available Maxpar metal‐tagged antibodies to realize the rapid identification of ROI with IFM using fluorescence signal, and then multiplexed detection of in situ ROI with IMC on the same tissue section.

## 2 Results and Discussions

### 2.1 MC‐Cdots(Ln) Synthesis and Characterization

**Figure 1:**
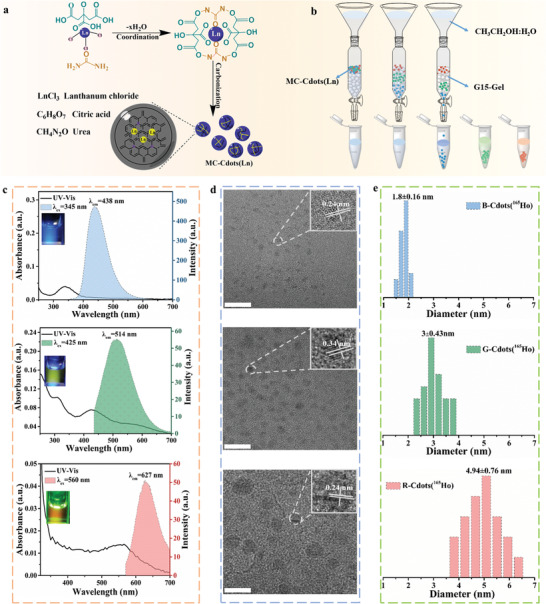
Synthesis and characterizations of MC‐Cdots(Ln). a) Synthesis pipeline of the MC‐Cdots(Ln). b) Separation of MC‐Cdots(Ln) using SEC column. c) UV–vis absorption and the fluorescence emission spectrum of Blue‐, Green‐, and Red‐Cdots(^165^Ho) at excitation wavelengths. Inset: photographs of Blue‐, Green‐, and Red‐Cdots(^165^Ho) solution under excitation wavelengths. d) TEM images of Blue‐ (upper), Green‐ (middle), and Red‐Cdots(^165^Ho) (bottom). Insets are the corresponding HRTEM, with a lattice parameter of 0.24 and 0.34 nm corresponding to (002) and (100) lattice plane, respectively. e) Size distributions of Blue‐, Green‐, and Red‐Cdots(^165^Ho). Abbreviations: MC‐Cdots(Ln), lanthanide‐doped multicolor fluorescent carbon nanodots; SEC: size exclusion chromatography column.

The MC‐Cdots(Ln) were synthesized by solvothermal approach (**Figure** **1**a), and the details are provided in the Experimental Section. The as‐prepared MC‐Cdots(Ln) showed excitation‐dependent PL properties with emission peaks ranging from 440 nm (blue) to 630 nm (red) at excitation from 340 to 580 nm (Figure S1, Supporting Information). Using Holmium (^165^Ho) as a model lanthanide, we fractionated the MC‐Cdots(^165^Ho) with Sephadex G‐15 SEC column as shown in Figure 1b. With ethanol‐water (1:1, v/v) as eluent, the blue‐, green‐ and red‐emission fluorescent fractions (named B‐, G‐, and R‐Cdots(^165^Ho), respectively) were obtained from raw MC‐Cdots(^165^Ho). The fluorescence emission peaks of B‐, G‐, and R‐Cdots(^165^Ho) were centered at 438, 514, and 627 nm, respectively (Figure 1c). The PL quantum yields (QY) of B‐, G‐, and R‐Cdots(^165^Ho) were 42.6%, 8.82%, and 6.92%, respectively. The purified fluorescent fractions exhibited excitation‐independent PL properties (Figure S2, Supporting Information). The transmission electron microscopy (TEM) images of B‐Cdots(^165^Ho), G‐Cdots(^165^Ho), and R‐Cdots(^165^Ho) showed a uniform and narrow size distribution with average diameters of about 1.8, 3, and 4.9 nm (Figure 1d,e). The high‐resolution TEM (HRTEM) images (insets of Figure 1d) further indicated that most of the carbon nanodots exhibited uniform atomic arrangements with high degree of crystallinity and a lattice parameter of 0.24 and 0.34 nm corresponding to (002) and (100) lattice plane, respectively. The gradually increased size of the purified fractions is consistent with the redshifted PL wavelength, which originates from the quantum confinement effect.

**Figure 2:**
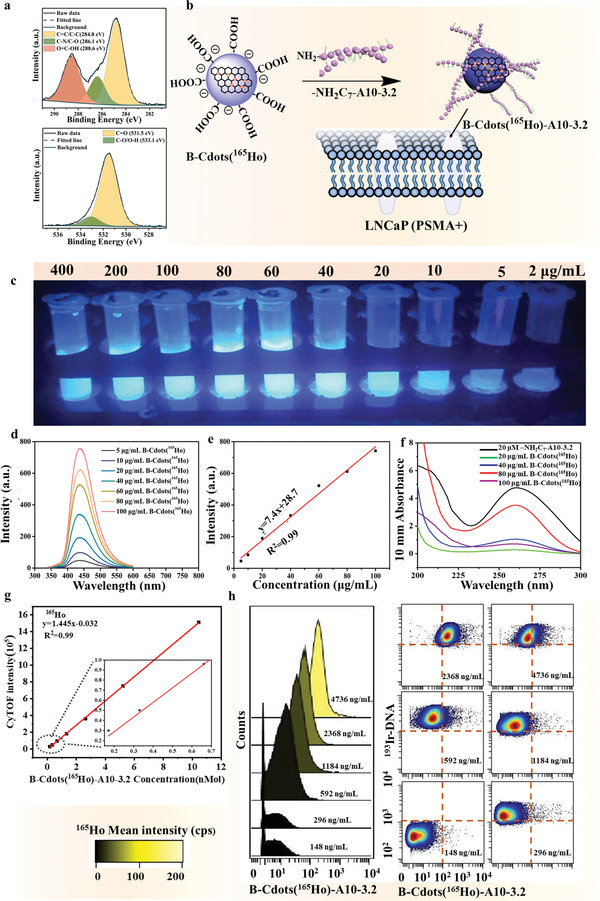
Synthesis and validation of B‐Cdots(^165^Ho)‐A10‐3.2. a) XPS high‐resolution of C 1s and O1s (MC‐Cdots(^165^Ho)). b) The B‐Cdots(^165^Ho)‐A10‐3.2 probe synthesized by amide reaction. c) Blue‐emitting fluorescence images of B‐Cdots(^165^Ho) at different concentrations. d) PL emission spectrum of B‐Cdots(^165^Ho) at various concentrations and e) its corresponding linearity curve. f) Typical absorption bands of B‐Cdots(^165^Ho)‐A10‐3.2 conjugates at different reaction concentration of B‐Cdots(^165^Ho). g) Dynamic range of B‐Cdots(^165^Ho)‐A10‐3.2 probe tested in solution‐mode measurements of mass cytometry (CyTOF). (Lanthanide element calibration beads were applied as normalization.) h) Titration histograms and contour plots of B‐Cdots(^165^Ho)‐A10‐3.2 against PSMA antigen with increasing concentrations of B‐Cdots(^165^Ho)‐A10‐3.2.

The urea and citric acid not only acted as the carbon sources for the MC‐Cdots(^165^Ho), but were also used to chelate with lanthanides through oxygen atoms to form a stable lanthanide‐doping carbon material. To test the generality of the preparation protocol for making other lanthanides‐doping carbon materials, seven different lanthanide ions, Ho^3+^, Tb^3+^, Tm^3+^, Ce^3+^, Lu^3+^, La^3+^, and Pr^3+^, were used to prepare MC‐Cdots(Ln). The lanthanide contents in Cdots were measured by inductively coupled plasma mass spectrometer (ICP‐MS) (Table S1, Supporting Information). The MC‐Cdots(^165^Ho), MC‐Cdots(^169^Tm), and MC‐Cdots(^159^Tb) showed the similar loading density of the lanthanide into the Cdots, which is essential for a panel of compatible functional mass tags. The surface functional carboxyl groups of the MC‐Cdots(^165^Ho), MC‐Cdots(^169^Tm), and MC‐Cdots(^159^Tb) were characterized by Fourier transform infrared (FTIR) spectroscopy (Figure S3, Supporting Information). Broad absorption bands at 3100–3500 cm^−1^ are assigned to *ν*(O—H) and *ν*(N—H). Absorption bands at 1600–1770 cm^−1^ are assigned to *ν*(C═O). These functional groups improve the hydrophilicity and stability of the MC‐Cdots(Ln) in aqueous system. The full‐scan X‐ray photoelectron spectroscopy (XPS) spectrum of MC‐Cdots(^165^Ho), MC‐Cdots(^169^Tm), and MC‐Cdots(^159^Tb) are shown in Figure S4 (Supporting Information). Taking MC‐Cdots(^165^Ho) as an example, four peaks at 284.6, 399.4, 530.9, and 128.2 eV are attributed to C1s, N 1s, O 1s, and Ho 4d XPS characteristic peaks. In the high‐resolution XPS spectra, the C 1s band can be fitted into three peaks, which reveals the presence of C═C/C—C (284.8 eV) and C—O/C—N (286.1 eV). Furthermore, the peak at 288.6 eV could be assigned to O═C—OH groups (**Figure** **2**a). The O 1s spectrum manifests that the peaks at 531.5 and 533.1 eV are for C═O and C—O/O—H, corroborating the presence of carboxylic groups on their surface (Figure 2a). Importantly, Cdots with —COOH/—CHO groups can be covalently linked with amine group containing molecules through an amide bond.^[^ ^20^ ^]^

### 2.2 Dual‐Functionality Probes Design and Optimization

The potential of B‐Cdots(^165^Ho) was evaluated by conjugating with A10‐3.2 aptamer against PSMA antigen for PaC cell lines (Figure 2b). Conjugation of B‐Cdots(^165^Ho) dual‐modal tag with —NH_2_C_7_ modified A10‐3.2 aptamer was performed by (1‐(3‐dimeth‐ylaminopropyl)‐3‐ethylcarbodimide hydrochloride‐N‐hydroxysuccinimide) (EDC‐NHS) covalent chemistry (Figure 2b), and the details are provided in the Experimental Section. According to the fluorescence‐emitting images of B‐Cdots(^165^Ho) aqueous solution at various concentration and corresponding PL emission spectra (Figure 2c–e), the fluorescence intensity of B‐Cdots(^165^Ho) at 20, 40, 60, 80, and 100 µg mL^−1^ is sufficiently strong to be used as fluorescence tags. Therefore, we optimized the degree of labeling with various concentrations of B‐Cdots(^165^Ho) (20, 40, 60, 80, and 100 µg mL^−1^) covalently linked to —NH_2_C_7_—A10‐3.2. The optical spectral properties of—‐NH_2_C_7_—A10‐3.2 aptamer and the synthesized B‐Cdots(^165^Ho)‐A10‐3.2 nanoprobe were investigated by a Nano‐100 Micro‐Spectrophotometer (Figure 2f). We found that the optimal concentration for B‐Cdots(^165^Ho) conjugating to —NH_2_C_7_—A10‐3.2 aptamer was 80 µg mL^−1^ (Figure 2f). Carbon dots with surface modifications are prone to aggregate in an aqueous solution if improper buffers and ionic strengths are applied. XPS has verified that functional carboxyl and amino groups exist on the surface of MC‐Cdots(^165^Ho), MC‐Cdots(^159^Tb), and MC‐Cdots(^169^Tm) tag. The high‐resolution C 1s, N 1s, and O 1s XPS spectra (Figure 2a and Figure S5, Supporting Information) confirm the existence of C═C/C—C (284.8 eV), C—O/C—N (286.1 eV), and C═O (288.8 eV) for C 1s; C═O (531.5 eV) and C—O/O—H (533.0 eV) for O 1s; pyrrolic N (398.2 eV), graphitic N (401.7 eV), and amine N (399.9 eV) for N 1s. Therefore, B‐Cdots(^165^Ho) at high concentration (100 µg mL^−1^) are more likely to aggregate by itself, which reduce reaction sites with the amino‐modified aptamers‐NH_2_C_7_‐A10‐3.2. On the other hand, the surface amino groups of carbon dots are involved in competitive reactions. As a result, the coupling reaction efficiency decreases at high concentrations of B‐Cdots (^165^Ho).The prepared B‐Cdots(^165^Ho)‐A10‐3.2 nanoprobe exhibited one absorption peak located at 260 nm, which is in accordance with the characterized absorption peak of —NH_2_C_7_—A10‐3.2. These results indicated that the basic structure of the —NH_2_C_7_—A10‐3.2 was not changed when conjugated to B‐Cdots(^165^Ho). To further confirm the successful conjugation of B‐Cdots(^165^Ho) to —NH_2_C_7_—A10‐3.2 aptamer molecular, the FTIR spectra of the B‐Cdots(^165^Ho) and B‐Cdots(^165^Ho)‐A10‐3.2 were first investigated, which was displayed in Figure S6 (Supporting Information). Compared with the B‐Cdots(^165^Ho), a typical absorption peak belonging to the —CONH— emerged at 1698 cm^−1^, which indicated the successful conjugation of aptamers via amide linkages. In addition, the B‐Cdots(^165^Ho)‐A10‐3.2 complexes showed characteristic absorption bands at 1050 cm^−1^ which could be assigned to the O—P stretching vibration of phosphate groups.^[^ ^21^ ^]^

To further determine the successful attachment of B‐Cdots(^165^Ho) to the aptamer molecule, we used solution‐mode measurements of mass cytometry (CyTOF) to measure the metal ^165^Ho signal of B‐Cdots(^165^Ho)‐A10‐3.2 probe, with element calibration beads (EQ beads) as normalization. Within the dynamic range of quantitative measurement of ^165^Ho, there is a linear regression between ^165^Ho signal intensities and B‐Cdots(^165^Ho)‐A10‐3.2 probe concentrations ranging from 0.2 to 10 × 10^−9^ m, as shown in Figure 2g. In the mass spectra, the mass spectrum peak of B‐Cdots(^165^Ho)‐A10‐3.2 (shown at the *m*/*z* of ^165^Ho) and other lanthanide elements are shown in Figure S7 (Supporting Information), suggesting that the aptamer‐based molecular probes were successfully synthesized. Molecular probe titration was further performed to validate and examine the specificity and affinity of the conjugated B‐Cdots(^165^Ho)‐A10‐3.2 molecular probe.^[^ ^22^ ^]^ B‐Cdots(^165^Ho)‐A10‐3.2 molecular probe was incubated with LNCaP cells at concentrations ranging from 148 to 4736 ng mL^−1^ in twofold dilutions. The specific calculation pipeline for concentrations of B‐Cdots(^165^Ho)‐A10‐3.2 probes is provided in the Experimental Section. Figure 2h shows the titration histograms and contour plots of B‐Cdots(^165^Ho)‐A10‐3.2 (with increasing concentration) against PSMA antigen. The binding of B‐Cdots(^165^Ho)‐A10‐3.2 to PSMA expressed on the LNCaP follows a saturation curve. These results demonstrated that there is only minimal nonspecific binding of B‐Cdots(^165^Ho)‐A10‐3.2 probe to target cells.

### 2.3 Dual‐Modal Imaging in PaC Cell Lines

**Figure 3:**
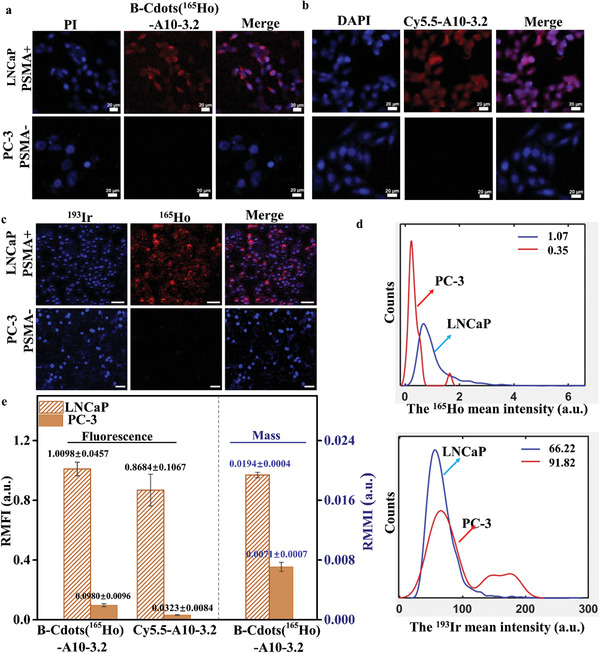
The B‐Cdots(^165^Ho)‐A10‐3.2 probe targeting to LNCaP and PC‐3 cells enable dual‐signal detection from IFM and IMC. a) IFM imaging of LNCaP and PC‐3 stained by B‐Cdots(^165^Ho)‐A10‐3.2. b) IFM imaging of LNCaP and PC‐3 stained by Cy5.5‐A10‐3.2 probe. (Pseudocolor red for B‐Cdots and Cy5.5 fluorescein, blue for PI.) c) IMC imaging of LNCaP and PC‐3 probed by B‐Cdots(^165^Ho)‐A10‐3.2. (Pseudocolor red for ^165^Ho‐PSMA, blue for ^193^Ir‐DNA.) d) The ^165^Ho and ^193^Ir mean intensity of LNCaP and PC‐3 cell lines. e) The relative mean fluorescence intensity (RMFI) as a ratio of fluorescent signals between B‐Cdots(^165^Ho)/Cy5.5(PSMA) and DAPI (nucleus), and the relative mean mass intensity (RMMI) as a ratio of the mean intensity between ^165^Ho (PSMA) and ^193^Ir (DNA‐intercalators) channels. Data are presented as mean ± SE (*n* = 5 replicates).

To verify the dual functionality of the B‐Cdots(^165^Ho)‐A10‐3.2, we performed staining experiments on human LNCaP (PSMA positive cell lines) and PC‐3 (PSMA negative cell lines) cells to assess whether B‐Cdots(^165^Ho)‐A10‐3.2 can be selectively localized to PSMA. Traditional ICC stain of LNCaP and PC‐3 (Figure S8, Supporting Information) with anti‐PSMA antibody (YPSMA‐1) was used as a standard control to define the distribution of PSMA on cells. The successful IMC imaging acquisition using MCPs(Ln)‐labeled A10‐3.2 aptamer probe has been previously demonstrated.^[^ ^23^ ^]^ From the fluorescence imaging results, the B‐Cdots(^165^Ho)‐A10‐3.2 stained LNCaP cells showed stronger fluorescence intensity compared to the negative PC‐3 cells (**Figure** **3**a), indicating that B‐Cdots(^165^Ho)‐A10‐3.2 showed effective specific targeting ability. The Cy5.5‐A10‐3.2 stained LNCaP and PC‐3 in a side‐by‐side experiment was used as control (Figure 3b).

The Cdots(^165^Ho)‐A10‐3.2 stained cells mounting on the glass slides were further scanned by IMC. Figure 3c shows the ^165^Ho and ^193^Ir channels, and merged images of LNCaP and PC‐3 cells. MCD Viewer software allows users to visualize MCD (.mcd) files generated by IMC. An open‐source, computational histology topography cytometry analysis toolbox (histoCAT) software uses a segmentation mask to extract the single‐cell data from the images.^[^ ^24^ ^]^ Such data include the abundance of the biomarkers on the ROI, cell size and shape. The mean intensity of ^165^Ho (corresponding to the abundance of PSMA expression) signal for the B‐Cdots(^165^Ho)‐A10‐3.2 stained LNCaP and PC‐3 was 1.07 and 0.35, respectively. The ^193^Ir (corresponding to the abundance of DNA) signal of the DNA‐intercalators stained LNCaP and PC‐3 are 66.22 and 91.82, respectively (Figure 3d). To eliminate instrument effects and for better quantification, we defined the relative mean mass intensity (RMMI) of ^165^Ho as the ratio of the mean intensities of the ^165^Ho and ^193^Ir channels. Therefore, the RMMI^165^_Ho_ for B‐Cdots(^165^Ho) stained LNCaP and PC‐3 are 0.0194 ± 0.0004 and 0.0071 ± 0.0007, respectively (Figure 3e). Similarly, the relative mean fluorescence intensity (RMFI) for B‐Cdots(^165^Ho) stained LNCaP and PC‐3 was calculated to be 1.0098 ± 0.0457 and 0.0980 ± 0.0096, respectively. Overall, B‐Cdots(^165^Ho)‐A10‐3.2 stained LNCaP cells express high levels of fluorescence and mass signals. No or little fluorescence and mass signal was detected from the negative‐control PC‐3 cells.

**Figure 4:**
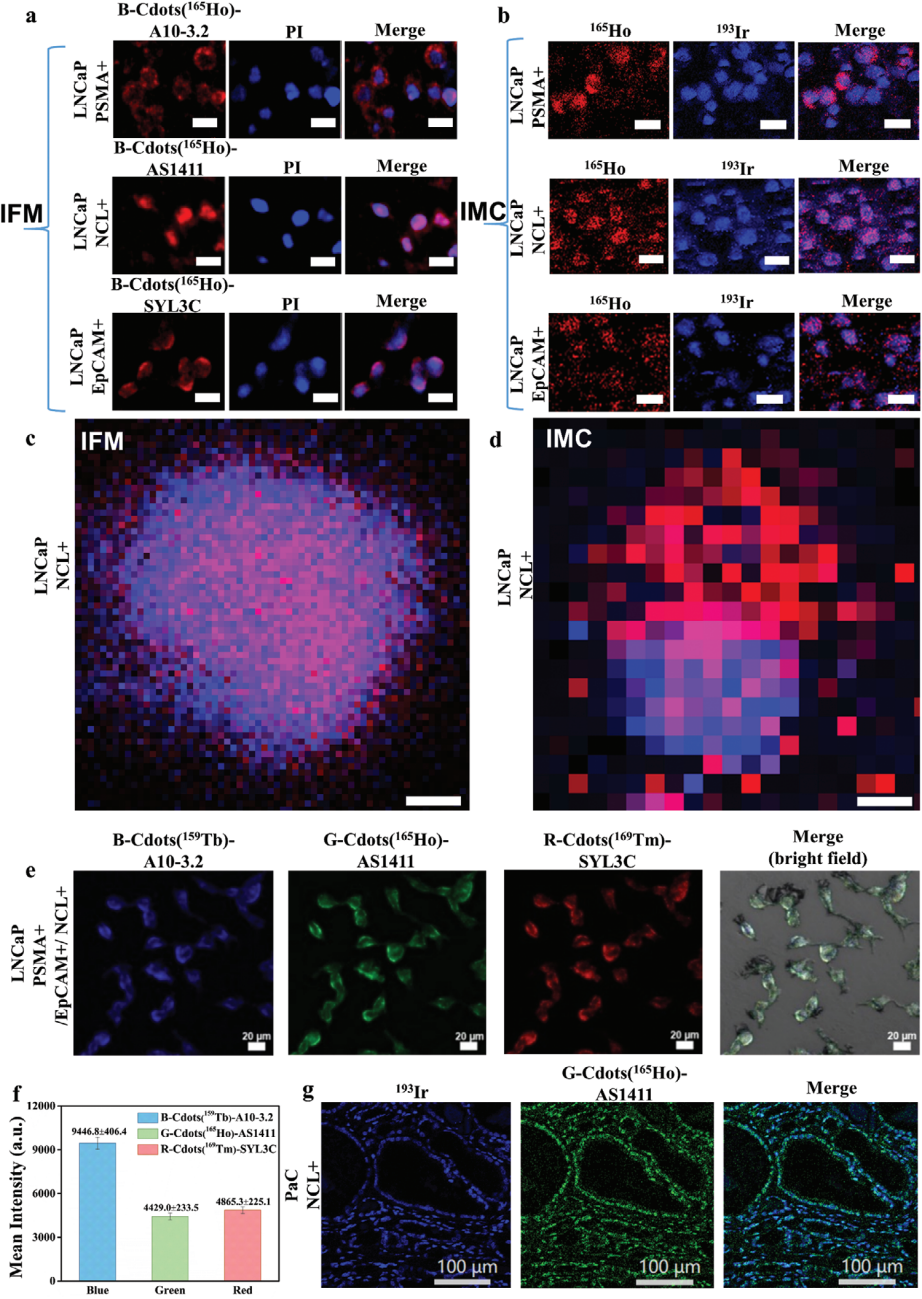
IFM provides more detailed spatial distribution of biomarkers comparing with IMC. a) Images of LNCaP stained by B‐Cdots(^165^Ho)‐A10‐3.2, B‐Cdots(^165^Ho)‐AS1411 and B‐Cdots(^165^Ho)‐SYL3C were scanned by IFM. b) Images of LNCaP stained by B‐Cdots(^165^Ho)‐A10‐3.2, B‐Cdots(^165^Ho)‐AS1411, and B‐Cdots(^165^Ho)‐SYL3C were scanned by IMC. The scale bar for (a) and (b) is 20 µm. c) Resolution of IFM image is 200 nm pixel^−1^ and d) IMC image is 1 µm pixel^−1^. The scale bar for (c) and (d) is 3 µm. e) IFM of LNCaP cell lines stained by B‐Cdots(^159^Tb)‐A10‐3.2, G‐Cdots(^165^Ho)‐AS1411, and R‐Cdots(^169^Tm)‐SYL3C, and the corresponding f) blue, green, and red mean fluorescence intensity. The mean intensity of blue‐, green‐, and red fluorescence of B‐Cdots(^159^Tb)‐A10‐3.2, G‐Cdots(^165^Ho)‐AS1411, and R‐Cdots(^169^Tm)‐SYL3C stained LNCaP are 9446.8 ± 406.4, 4429.0 ± 233.5, and 4865.3 ± 225.1, respectively. Data are presented as mean ± SE (*n* = 5 replicates). g) The PaC IMC images of ^193^Ir‐DNA‐intercalators (blue), ^165^Ho‐NCL (green), and their overlay.

We then extended aptamer‐based molecular probes to form a library of dual‐functional probes. SYL3C and AS1411 are DNA‐based aptamers, which can specifically bind to EpCAM and NCL.^[^ ^25^, ^26^ ^]^ EpCAM is an important type I transmembrane protein that is overexpressed on the surfaces of most cancer cells and involved in cell adhesion and cell signaling.^[^ ^27^ ^]^ NCL is a multifunctional protein that mainly localizes in the nucleolus and distributes in the nucleoplasm, cytoplasm, and cell membrane.^[^ ^28^ ^]^ We used B‐Cdots(^165^Ho)‐SYL3C and B‐Cdots(^165^Ho)‐AS1411 aptamers to label EpCAM and NCL proteins of LNCaP cells, respectively, and used IFM and IMC to investigate the distribution of EpCAM and NCL (**Figure** **4**a,b). The IFM provided the more detailed and accurate spatial resolution for these functional proteins at optical resolution (200 nm pixel^−1^) (Figure 4c), compensating for the low resolution of the IMC imaging (1 µm pixel^−1^) (Figure 4d). We then labeled LNCaP cells and PaC tissues with B‐Cdots(^159^Tb)‐A10‐3.2, G‐Cdots(^165^Ho)‐AS1411, and R‐Cdots(^169^Tm)‐SYL3C aptamers and demonstrated the imaging of three biomarkers simultaneously with high resolution and specificity (Figure 4e,g and Figure S9, Supporting Information). The specific fluorescence intensity for B‐Cdots(^159^Tb)‐A10‐3.2, G‐Cdots(^165^Ho)‐AS1411, and R‐Cdots(^169^Tm)‐SYL3C stained LNCaP are 9446.8 ± 406.4, 4429.0 ± 233.5, and 4865.3 ± 225.1, respectively (Figure 4f).

Next, we have studied the stability of probes by measuring the Ln^3+^ leakage from the probes through ICP‐MS. A certain volume of purified B‐Cdots(^159^Tb)‐A10‐3.2, G‐Cdots(^165^Ho)‐AS1411, and R‐Cdots(^169^Tm)‐SYL3C aqueous solution were loaded inside the Float‐A‐Lyzer G2 dialysis devices (MWCO: 0.5–1.0 kD, 1 mL), and the sample was dialyzed at room temperature for 4 d (24–96 h) in DEPC‐treated water. A small volume of sample was aspirated out (at 12 h intervals) from the inside and outside of dialysis bag for Ln^3+^ content detection. From the ICP‐MS results, the MC‐Cdots(Ln)‐aptamers showed no significant Ln^3+^ leakage into DEPC‐treated water and almost no Ln^3+^ content change in dialysis bag, indicating good stability (Figure S10, Supporting Information).

In addition, to further verify the cytotoxicity of the B‐Cdots(^159^Tb)‐A10‐3.2, G‐Cdots(^165^Ho)‐AS1411, and R‐Cdots(^169^Tm)‐SYL3C synthetic probes, the cytotoxicity assay was investigated by the Cell Counting Kit‐8 (CCK‐8) assay. LnCaP and PC‐3 cells were seeded (5000 cells per well) in 96‐well plates and grew overnight. And then, the cells were replaced with fresh medium containing different concentration (2.5–400 ng µL^−1^) of B‐Cdots(^159^Tb)‐A10‐3.2, G‐Cdots(^165^Ho)‐AS1411, and R‐Cdots(^169^Tm)‐SYL3C probes. After incubation for 24 h, the CCK‐8 reagent (10%) was added to each well to test the cell viability through absorbance measurement at a wavelength of 450 nm with a microplate reader (BioTek). As shown in Figure S11 (Supporting Information), all of the survival rates of the cells exceed ≈85% after incubation with B‐Cdots(^159^Tb)‐A10‐3.2 and R‐Cdots(^169^Tm)‐SYL3C probes for 24 h at six different concentrations (2.5–400 ng µL^−1^). However, the cell survival rate decreased to 75% after incubation with G‐Cdots(^165^Ho)‐AS1411 probe for 24 h at the concentration of 40 ng µL^−1^, which may be caused by the antineoplastic properties of the AS1411 aptamer itself.^[^ ^29^ ^]^ These results indicate that B‐Cdots(^159^Tb)‐A10‐3.2, G‐Cdots(^165^Ho)‐AS1411, and R‐Cdots(^169^Tm)‐SYL3C probes do not induce significant inhibition toward the proliferation of the cells.

### 2.4 PaC Tissue Sections IFM Location and IMC Deep‐Profiling Analysis

**Figure 5:**
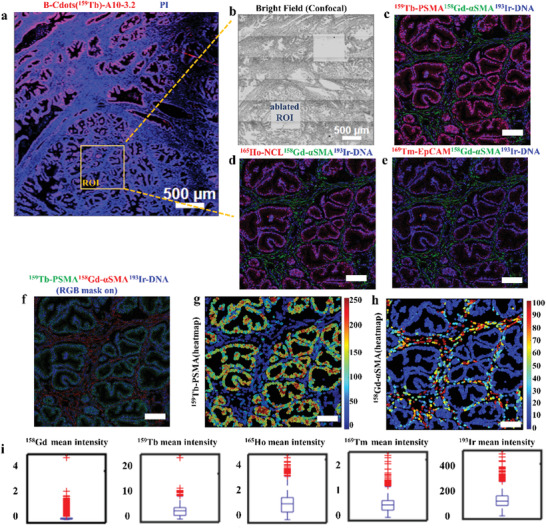
PaC tissue section was stained with a hybrid panel of B‐Cdots(^159^Tb)‐A10‐3.2, G‐Cdots(^165^Ho)‐NCL, R‐Cdots(^169^Tm)‐SYL3C probes, and MaxPar X8 metal‐tagged antibodies, and then sequentially analyzed by IFM and IMC. a) ROI was selected by IFM. b) The bright‐field image indicates the overall tissue section after laser ablating the selected ROI. Overlay of c) PSMA (red), *α*‐SMA (green), and DNA (blue), d) NCL (red), *α*‐SMA (green), and DNA (blue), e) EpCAM (red), *α*‐SMA (green), and DNA (blue) is co‐visualized. The scale bar for (c)–(e) is 100 µm. f) Overlay of segmentation mask on the images. The heatmap image of g) ^159^Tb channel and h) ^158^Gd channel is displayed. i) The mean intensity of ^158^Gd‐*α*‐SMA, ^159^Tb‐PSMA, ^165^Ho‐NCL, ^169^Tm‐EpCAM, and ^193^Ir‐ DNA‐intercalators is acquired.

We then mixed the synthetic B‐Cdots(^159^Tb)‐A10‐3.2, G‐Cdots(^165^Ho)‐AS1411, and R‐Cdots(^169^Tm)‐SYL3C dual‐functional probes with the conventional Maxpar X8 metal‐tagged antibodies for further analyzing human formalin‐fixed, paraffin‐embedded (FFPE) PaC tissue. The panel included three dual‐functional aptamer‐based probes (PSMA, EpCAM (epithelial marker), nucleolin (NCL, proliferation marker)), and seven Maxpar antibodies. The Fluidigm Maxpar X8 metal‐labeled antibodies are as below: E‐cadherin (E‐CAD, epithelial marker), *α*‐smooth muscle actin (*α*‐SMA, stromal), cytokeratin7 (CK‐7, luminal marker) structural proteins, platelet endothelial cell adhesion molecule‐1 (PECAM‐1/CD31) expressed on endothelial cells, CD44 (cancer stem cell marker, adhesion), CD45 (leukocyte marker), and single‐chain type‐1 glycoprotein (MIC2/CD99). CD99 is a heavily O‐glycosylated transmembrane protein (32 kDa) expressed on leukocytes and activated endothelium (**Table** **1**). Maxpar X8 metal‐labeled antibodies had been previously validated in the IMC systems. The sample preparation is similar to standard IHC protocols, where the FFPE tissue section on a slide is first deparaffinized and treated with an antigen retrieval buffer for antigen epitope exposure, then stained with a mixture of metal‐labeled antibodies and B‐Cdots(^159^Tb)‐A10‐3.2, G‐Cdots(^165^Ho)‐AS1411, and R‐Cdots(^169^Tm)‐SYL3C dual‐functional probes. In the tissue section dual‐modal imaging, a two‐step procedure was used. The tissue section was first scanned with IFM to quickly identify the ROI using fluorescence signal of probes and then sequentially interrogated with the panel of metal‐labeled antibodies and aptamers for deep proteomic profiling with IMC. An area of 3.5 mm × 3.5 mm fluorescent image of the PaC tissue sections was recorded by tile scanning mode of IFM (**Figure** **5**a), and the scanning process was quickly accomplished within 180 s. The selected ROI on the same tissue section was then realized multiplexed detection by IMC (Figure 5b). Notably, an area of 3.5 mm × 3.5 mm image typically requires ≈150 h blind laser scanning by IMC, and with the presented pipeline using dual‐mode probes, the overall acquisition time for 3.5 × 3.5 mm image could save up to about 90%. As shown in Figure 5a, the distribution of PSMA‐positive epithelial cells in the IMC images is consistent with that observed in the IFM image. In addition, the ^159^Tb, ^165^Ho, and ^169^Tm channels provided by dual‐functional probes did not encounter obvious channel contamination to Maxpar X8 isotopic mass channels (^158^Gd‐*α*‐SMA, ^168^Er‐E‐CAD, ^145^Nd‐CD31, and ^161^Dy‐CD44) (Figure 5c–e and Figure S12, Supporting Information). We further analyzed the PSMA and *α*‐SMA intensity distribution through the heatmap using histoCAT. The heatmap quantified the PSMA and *α*‐SMA expression level with topological information (Figure 5g,h). The heatmap of the ^159^Tb and ^158^Gd channels suggested that the metal probes specifically targeted the prostate acinar epithelial cells and stromal cells on the PaC tissue. Image‐derived biomarker intensity was extracted from histoCAT and shown in Figure 5i. Our results indicated the PSMA content was significantly overexpressed compared to the other cancer biomarkers on PaC tissue section.

**Table 1:** Hybrid panel of Maxpar X8 metal‐tagged antibodies and MC‐Cdots(Ln) labeled aptamers for multiplex PaC tissue section imaging

Reagent	Name‐metal	Clone	Marker	Vendor
Maxpar antibodies	*α*‐SMA‐^158^Gd	1A4	Stromal	Abcam
	CK7‐^175^Lu	RCK105	Luminal/epithelial	Abcam
	E‐CAD‐^168^Er	4A2	Epithelial	Abcam
	CD31‐^145^Nd	JC/70A	Endothelial	Novus Bio
	CD44‐^161^Dy	CA4Mab‐5	Adhesion	Biolegend
	CD45‐^150^Nd	HI30	Immune	Biolegend
	CD99‐^172^Yb	3B2/TA8	Endothelial	Biolegend
MC‐Cdots(Ln) aptamers	PSMA‐B‐Cdots‐^159^Tb	–	Proliferating	Sangon Biotech
	NCL‐G‐Cdots‐^165^Ho	–	Epithelial	Sangon Biotech
	EpCAM‐R‐Cdots‐^169^Tm	–	Epithelial	Sangon Biotech
Cell identification	DNA intercalator‐^191^Ir/^193^Ir	–	Nuclei	Fluidigm

**Figure 6:**
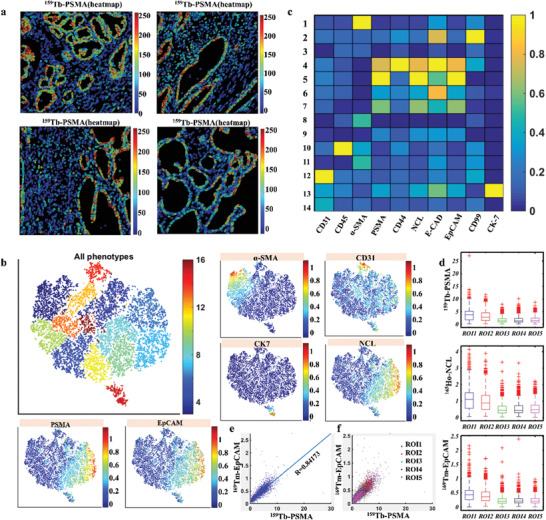
histoCAT analysis of cell subtypes in high‐dimension images of PaC tissue sections. a) PSMA distributions in different ROI of multiple PaC tissue sections indicate universal reliability and affinity of the dual‐functional probes. b) PhenoGraph defines complex cell phenotypes based on marker expression and enables labeling of cell phenotype clusters on a t‐SNE plot. c) Heatmap of biomarkers expressing on different cell clusters. d) Mean intensity of PSMA, NCL, and EpCAM biomarkers expressing on different ROI as shown in boxplots. e,f) Linear correlation and consistency of PSMA and EpCAM expression level are observed.

We further analyzed targeted images that were collected from five diverse ROI. The ^159^Tb channel heatmap of diverse ROI indicated that the synthesized B‐Cdots(^159^Tb)‐A10‐3.2 probe specifically targeted the prostate acinar epithelial cells of different ROI (**Figure** **6**a) and suggested the desired stability of the probes. To gain a tissue‐wide overview of cell phenotypes present in a given image sets, we visualized the image sets using tSNE, a dimensionality reduction method that projects high‐dimension cell data into two dimensions. PhenoGraph is an unsupervised clustering algorithm incorporated into histoCAT. In the PaC cancer tissue samples, PhenoGraph identified 14 phenotype clusters (PC) shared across images, and these clusters were then visualized on a tSNE map (Figure 6b). These cell phenotypes are present at different frequencies and characterized by specific epitopes (e.g., *α*‐SMA is specific for PC 1, and CD31 is specific for PC 12) and combinations of markers (e.g., proliferative NCL for PCs 4 and 5) (Figure 6b). On the tSNE map in histoCAT, expression of individual markers can be highlighted using color scales (Figure 6c). The ^159^Tb, ^165^Ho, and ^169^Tm channel mean intensity, corresponding to the expression level of PSMA, NCL and EpCAM proteins in different ROI, are shown in boxplots (Figure 6d). The high PSMA expression level is consistent with the PaC PSMA positive cell lines. In addition, the scatter plots of PSMA and EpCAM indicated that the expression level is linearly positive (Figure 6e,f).

## 3 Conclusions

We developed a new class of multicolor lanthanide‐doped fluorescent carbon nanodots (MC‐Cdots(Ln)) dual‐functional tags and demonstrated their use for labeling aptamers as fluorescence and mass dual‐imaging probes. We have mixed the synthetic dual‐functional MC‐Cdots(Ln)‐aptamers probes with commercially available MCPs(Ln) labeled antibodies probes, and then realized the compatible co‐staining on the PaC tissue section. The prepared MC‐Cdots(Ln)‐aptamers dual‐functional probes could localize the ROI promptly through fluorescence signal (3.5 mm × 3.5 mm needs only ≈180 s), and the marked ROI was sequentially allowed for further multiplexed proteomic analysis by IMC. The overall process time was saved up to about 90% compared to IMC blind scanning. Furthermore, the dual‐functional probes also protected serial sections of rare precious clinical tissue samples from inaccurate dislocation. Notably, the MC‐Cdots(Ln)‐aptamers dual‐functional probes did not cause obvious channel contaminations to commercial Maxpar antibodies. The capability of simultaneous data acquisition on IFM and IMC enabled advanced imaging synthetically with rapid speed, high multiplicity, and refined resolution, which harmonized the issues encountered in either technology used alone. However, the clinical applications of the dual‐functional probes were restricted by the limited fluorescence channels (B‐, G‐, R‐fluorescence) due to broad absorption and emission spectra of carbon dots. Efforts are underway to extend the synthesis and purification protocols to multicolor lanthanide‐doped carbon dots with high fluorescence quantum yield for multiplexed dual‐modal imaging.

## 4 Experimental Section

### Chemicals and Materials

Citric acid (CA, C_6_H_8_O·H_2_O) and carbonyldiamine (Urea, CH_4_N_2_O) were obtained from Sinopharm Chemical Reagent Co., Shanghai, China and Shanghai Yuanye Bio‐Technology Co., Shanghai, China, respectively. DMF was purchased from Inno‐Chem Technology Co., Beijing, China. Terbium nitrate pentahydrate (Tb(NO_3_)_3_·6H_2_O), thulium nitrate hexahydrate (Tm(NO_3_)_3_·6H_2_O), cerium nitrate pentahydrate (Ce(NO_3_)_3_·6H_2_O), holmium nitrate pentahydrate (Ho(NO_3_)_3_·6H_2_O), lanthanum nitrate pentahydrate (La(NO_3_)_3_·6H_2_O), praseodymium nitrate pentahydrate (Pr(NO_3_)_3_·6H_2_O), and lutetium nitrate pentahydrate (Lu(NO_3_)_3_·6H_2_O) were purchased from Saen Chemical Technology Co., Shanghai, China. CCK‐8 was purchased from Dojindo Molecular Technologies (Japan), and 20× DEPC treated phosphate‐buffered saline (PBS) buffer (pH 7.2–7.6) was purchased from Sangon Biotech (Shanghai) Co., Ltd. All of the reagents were analytically pure and used without any purification. Sephadex G‐15 gel was obtained from Shanghai Macklin Biochemical Co., Shanghai, China. The —NH_2_C_7_ modified A10‐3.2 aptamer: (—NH_2_C_7_‐5′‐GGG AGG ACG AUG CGG AUC AGC CAU GUU UAC GUC ACU CCU‐3′), —NH_2_C_7_ modified AS1411 aptamer (—NH_2_C_7_‐5′‐GGT GGT GGT GGT TGT GGT GGT GGT GGT TTT TT‐3′), —NH_2_C_7_ modified SYL3C aptamer: (—NH_2_C_7_‐5′‐CAC TAC AGA GGT TGC GTCT GTC CCA CGT TGT CAT GGG GGG TTG GCC TG‐3′), and 3′‐NH_2_ modified control oligonucleotides (ctDNA, NH_2_C_7_‐5′‐CCT CCT CCT CCT TCT CCT CCT CCT CCT TTT TT‐3′) were synthesized and purified by Shanghai Sangon Biotechnology Co. Ltd.

### Equipments

TEM images were recorded with TALOS F200X (FEI Co., USA) operated at an accelerating voltage of 200 kV. XPS analysis was performed with a Kratos Axis Ultra DLD spectrometer (Shimadzu, Tokyo, Japan) using a monochromated Al Ka X‐ray source (*hν* = 1486.6 eV). Ultraviolet–visible (UV–vis) spectra were collected using a Cary 60 UV–vis spectrophotometer (Agilent Technologies). The PL measurements were performed on a Cary Eclipse Fluorescence Spectrophotometer (Agilent Technologies). Fluorescence images of cells and tissues sections were acquired by Zeiss LSM 880 confocal microscope. The Ln^3+^ content was measured by ICP‐MS (I CAP Q, Thermo Co., USA). The cell viability was tested by microplate reader (BioTek).

### Preparation of MC‐Cdots(Ln)

MC‐Cdots(Ln) were first synthesized by solvothermal procedure. Briefly, 1 g of urea and 0.2 g of lanthanide salt first dissolved in 10 mL of DMF, and reacted for 1 h at room temperature. Subsequently, 0.5 g citric acid was added to the solution described above. After complete dissolution, the mixture was transferred to a 30 mL Teflon‐lined stainless‐steel autoclave and heated at 200 °C for 4 h. After the reaction, the autoclaves were cooled to room temperature naturally. The resulted dark brown and transparent solution was centrifuged at 4000 rpm for 20 min to remove large nonfluorescent particle first. Then, the suspension was centrifuged twice (15 000 rpm for 2 h, 4 °C) with anhydrous ethanol to wash off superfluous organic molecule. The precipitation was next suspended in deionized water and dialyzed against deionized water with MWCO = 1000 Da for 7 d to remove the salt and finally obtain MC‐Cdots aqueous solution, which could be freeze‐dried to give the dark powder products.

### Separation of MC‐Cdots(Ln) into Blue, Green, and Red Fluorescent Fractions

MC‐Cdots(Ln) were separated by column chromatography on a Sephadex G‐15 gel filtration column using water‐ethanol (1:1, v/v) as the eluent. The gel (5 g) was swollen in eluent for 6 h, and the supernatant (including the suspended ultrafine gel) was discarded for the preparation of the column. Slurry media were settled in a ratio of 75% gel to 25% eluent and degassed ultrasonically. A glass column (13 mm inner diameter) was filled with eluent to remove air bubbles and then closed. Flush the column with eluent, leaving a few milliliters at the bottom, the column was then opened for the continuous addition of the gel suspension. The gel‐filled column was washed until no change in height (12 cm) was observed. The MC‐Cdots(Ln) aqueous solution was then added to the gel column and eluted with the eluent. The wavelength of 302 nm was used to monitor the specimens eluting from the column, and the eluted solution was collected for further use. As a result, three fractions of Cdots, named B‐, G‐, and R‐Cdots(Ln), were collected according to their PL properties.

### Quantum Yield Measurements

An established relative method, using quinine sulfate in 0.10 m H_2_SO_4_ solution as the fluorescence standard to determine the quantum yield of three fluorescent fractions. According to the established procedure, the UV absorbance and the fluorescence emission spectrometry of samples and standard substance were tested. The quantum yields of B‐Cdots(Ln), G‐Cdots(Ln), and R‐Cdots(Ln) were estimated according to the following equation

(1) $$Q_{\mathrm{Cdots}}=Q_{\mathrm{quinine}}\;\times \frac{A_{\mathrm{quinine}}}{A_{\mathrm{Cdots}}}\times \frac{F_{\mathrm{Cdots}}}{F_{\mathrm{quinine}}}\times \frac{n_{\mathrm{Cdots}}^{2}}{n_{\mathrm{quinine}}^{2}}$$

where *Q* is the quantum yield, *A* is the absorbance, *F* is the integrated fluorescence intensity, and *n* is the refractive index. The subscript refers to the Cdots sample or the reference fluorophore, quinine sulfate solution. To minimize reabsorption effects, the UV absorbances of Cdots and quinine sulfate were kept below 0.1.

### Fabrication of MC‐Cdots(Ln)‐Aptamer Conjugates

MC‐Cdots(Ln) suspension (80 µg mL^−1^, 60 µL) was first activated by mixing with 10 µL of 6.25 mol L^−1^ EDC and 10 µL of 6.25 mol L^−1^ NHS in PBS (pH 7.2–7.6, Nuclease free). After incubation at 37 °C for 30 min, 20 µL of 5′‐NH_2_C_7_ modified aptamer (20 µmol L^−1^ in DNase RNase‐free water) was added to the mixture and incubated for an additional 4 h at 37 °C. After conjugation, the reaction system was transferred to 10 kDa spin filter, and the free aptamer and MC‐Cdots(Ln) were removed at 15 000 rpm for 10 min at 4 °C. The purified MC‐Cdots(Ln)‐aptamer conjugates were stored at 4 °C for latter experiments.

### PaC Cell Lines Cultivation and Mounted on Glass Slides

LNCaP and PC‐3 cells were cultured in Dulbecco's modified Eagle's medium (DMEM) containing penicillin (100 U mL^−1^), streptomycin (100 U mL^−1^), and 10% fetal bovine serum (FBS) in a humidified incubator at 37 °C and 5% CO_2_. The cells were digested by trypsin and resuspended in fresh complete medium before plating. Each cell line was seeded onto individual‐chamber slides pretreated with polylysine, at a density of 1 × 10^5^ cells mL^−1^ in 300 µL of medium. After culturing for 24 h at 37 °C and 5% CO_2_, the medium was sucked out and cells were rinsed in PBS. Afterward, the cells were fixed in 4% PFA for 10 min, washed in PBS, and stored at −20 °C.

### B‐Cdots(^165^Ho)‐A10‐3.2 Conjugates Used for CyTOF Titration Process

LNCaP and PC‐3 cells (no more than 10^7^ cells per tube) were collected by centrifugation and resuspended in 1 mL FBS free RPMI‐1640 preheated at 37 °C. For live/dead cell discrimination, 1 µL of 5 × 10^−3^ m cisplatin (Fluidigm) was added to each tube and mixed well. After incubation in 37 °C water bath for 5 min, cisplatin was quenched by addition of 5 mL cell staining buffer (CSB, 1× PBS, 0.5% BSA, 0.02% NaN_3_), and cells were centrifuged down (300×*g*, 5 min, room temperature) and supernatants aspirated. Then, cells were resuspended in 1 mL CSB and fixed by adding fixation solution (3.2% paraformaldehyde in PBS, 1 mL) drop by drop and vortex blending immediately. After incubation for 10 min at room temperature, 4 mL of CSB precooled at 4 °C was added to slow fixation, and then cells were centrifuged, resuspended, and counted. Finally, the fixed cell pellets were stored in 10% DMSO/CSB at −80 °C.

The PFA fixed, frozen cells were firstly removed from the −80 °C refrigerator and melted on ice or in a cold water bath, and then added to six FACS tubes (1 × 10^6^ LNCaP cells each tube). The cell pellets were centrifuged with 2 mL CSB and supernatants aspirated. Then the cell suspensions were stained with a gradient dilution of tagged aptamer (MC‐Cdots(^165^Ho)‐A10‐3.2) at room temperature for 30 min. The concentrations of probes were calculated by the following formula

(2) $$\mathrm{Concentratio}n_{\mathrm{probe}}=\frac{\eta \times C_{0}\times V_{0}\times M_{w}}{V_{\mathrm{probe}}}\;$$

*η*: The coupling efficiency of B‐Cdots(^165^Ho) with —NH_2_C_7_—A10‐3.2 aptamer
*C*_0_: The molar concentration of —NH_2_C_7_—A10‐3.2 of the conjugation reaction, µmol L^−1^
*V*_0_: The reaction volume of —NH_2_C_7_—A10‐3.2, µL
*M*_w_: Molecular mass of —NH_2_C_7_—A10‐3.2, g mol^−1^
*V*_probe_: The volume of B‐Cdots(^165^Ho)‐A10‐3.2, µL.

After being washed with CSB twice (500×*g*, 5 min, room temperature), the cells were incubated with DNA Intercalator‐Ir (125 × 10^−9^ m) for 1 h at room temperature to identify nucleated events. Finally, samples were washed with CSB once and deionized water three times, and dispersed onto deionized water containing 10% EQ beads before the injection into the mass cytometer.

### Staining of PaC Cell Lines Mounted on Slides for IFM and IMC

The LnCAP and PC‐3 cell lines mounted on the slides were stained with MC‐Cdots(Ln)‐aptamer: First, the cells mounted on the chamber slides were washed twice in PBS, permeabilized for 5 min in 0.1% Triton X‐100 and washed again in PBS for three times. After being blocked with 3% BSA in PBS for 30 min at room temperature, the cells were incubated overnight with the probes at 4 °C in hydration chamber. After that, the slides were washed three times in DPBS. Finally, the cells were stained with the nuclear dye (DAPI or PI) for fluorescent imaging; Cell‐ID DNA Intercalator‐Ir for imaging mass cytometry, and then rinsed in doubly distilled water.

### Staining of Prostate Tissue Sections for IFM and IMC

The FFPE tissue sections were pretreated using standard protocols before staining. First, the tissue sections were heated in the oven at 60 °C for 2 h and then immediately dewaxed in xylene for 20 min. These slides were subsequently rehydrated in descending grades of ethanol aqueous solution (100%, 95%, 80%, 70%, deionized water; 5 min each). Then antigen retrieval was performed in an autoclave sterilizer high‐pressure sterilizer at 96 °C in sodium citrate buffer (pH 9, antigen retrieval solution 1×) for 30 min. After being cooled to below 70 °C within 10 min, tissue pieces were washed with deionized water and DPBS followed.

After antigen retrieval, the FFPE cancer tissue slides were first blocked with 3% BSA in PBS for 45 min at room temperature, and then incubated with MC‐Cdots(Ln)‐aptamers mixing with polymer X8 labeled antibodies probes in 0.5% BSA/DPBS overnight at 4 °C in hydration chamber, followed by washing with 0.1% Triton X‐100/DPBS and PBS (8 min, twice each). The nuclei were stained with DAPI for 5 min and DNA Intercalator‐Ir for 30 min at room temperature. Slices were washed with PBS, rinsed with deionized water for 5 min after DAPI or DNA Intercalator‐Ir staining, and then dried 20 min at room temperature. All the formalin‐fixed and paraffin‐embedded tissue sections were approved by the Human Ethics Review Committee of Science and Technology, Shanghai Jiao Tong University according to the Chinese regulation.

### Statistical Analysis

Cell events were collected on Helios time‐of‐flight mass cytometer (CyTOF, Fluidigm 108001), and data analysis was performed using the Cytobank platform (www.cytobank.org) as previously described.^[^ ^30^ ^]^ EQ bead signals were used to normalize the signal fluctuations of Helios prior to data export and analysis.^[^ ^31^ ^]^ The signal intensity values collected by CyTOF were transformed by a scaled arcsinh with a factor of 5, which diminished noise values in the measurements. The IMC data were acquired from the Hyperion imaging system (Fluidigm 108001; laser type, Nd:YAG, 213 nm; ablation spot size, 1 µm^2^; 135 channels, 75–209 amu) and analyzed by MCD Viewer (Fluidigm), CellProfiler (Carpenter Lab, Broad Institute, MIT), and histoCAT (Bodenmiller lab, Institute of Molecular Life Sciences, University of Zurich) software. The TEM size distributions of blue‐, green‐, and red‐Cdots(^165^Ho) dual‐functional tags were analyzed by ImageJ software. Origin 2019b was used to calculate mean and standard error of the mean, and the results were expressed as means ± SE value. No statistical methods were used to predetermine sample sizes, and the exact values of *n* (sample size) are provided in each figure legends.

## Conflict of Interest

The authors declare no conflict of interest.

## Supporting information

Supporting Information

## Data Availability

Data are available on request from the authors.
