# Supplementary material for: Aptamer Probes Labeled with Lanthanide‐Doped Carbon Nanodots Permit Dual‐Modal Fluorescence and Mass Cytometric Imaging
Source: Adv Sci (Weinh). 2021 Nov 1;8(24):2102812. doi: 10.1002/advs.202102812 (PMC8693039; doi:10.1002/advs.202102812)
Supplement: Supplementary file 1 — Supporting Information [file ADVS-8-2102812-s001.pdf]

© 2021 Wiley-VCH GmbH

ADVANCED SCIENCE
Open Access

Supporting Information

for *Adv. Sci.,* DOI: 10.1002/advs.202102812

Aptamer Probes Labeled with Lanthanide-Doped Carbon Nanodots permit Dual-Modal Fluorescence and Mass Cytometric Imaging

*Youyi Yu, Xin Wang, Xiaolong Jia, Zijian Feng, Lulu Zhang, Hongxia Li, Jie He, Guangxia Shen**, and *Xianting Ding**

Aptamer Probes Labeled with Lanthanide-Doped Carbon Nanodots permit Dual-Modal Fluorescence and Mass Cytometric Imaging

Youyi Yu‡1, Xin Wang‡1, Xiaolong Jia[2], Zijian Feng[1], Lulu Zhang[1], Hongxia Li[1], Jie He[1], Guangxia Shen*1, Xianting Ding*1

1 State Key Laboratory of Oncogenes and Related Genes, Institute for Personalized Medicine, School of Biomedical Engineering, Shanghai Jiao Tong University, Shanghai, 200030, China.

2 Department of Urology, Ningbo First Hospital, Ningbo Hospital of Zhejiang University, Ningbo, Zhejiang Province, China.

‡These authors contributed equally.

* gxshen@sjtu.edu.cn; dingxianting@sjtu.edu.cn

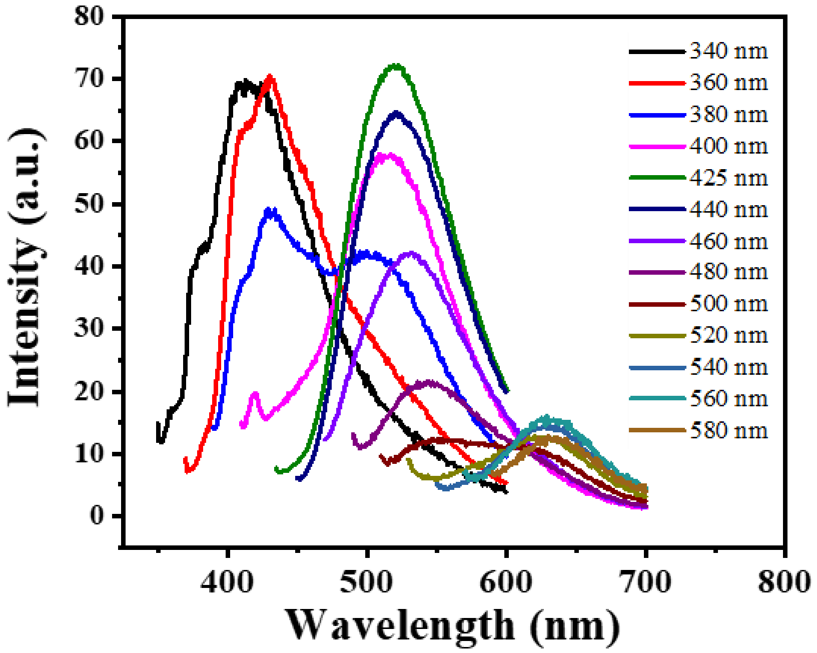

Figure S1 Photoluminescent spectra of MC-Cdots(^{165}Ho) (0.35 mg/mL) under different excitation wavelengths.

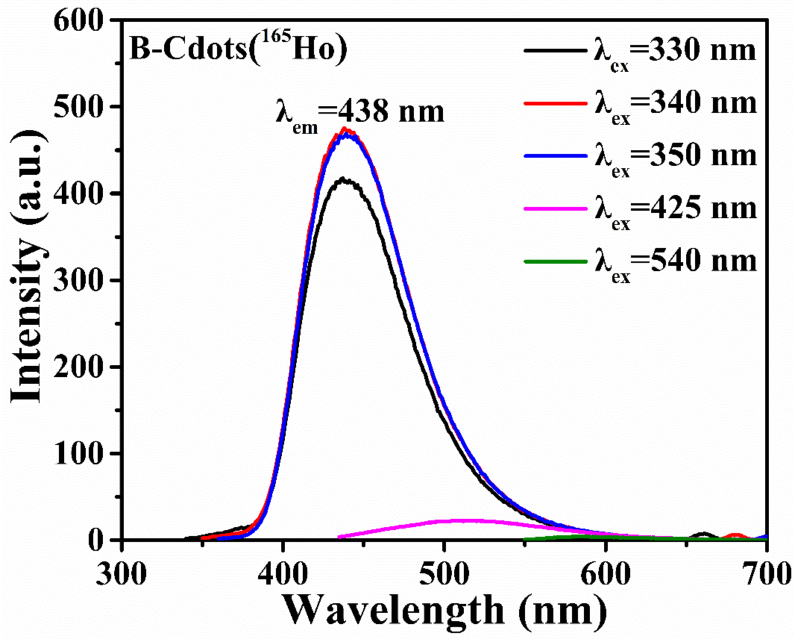
B-Cdots(^{165}Ho)
λ_{em}=438 nm
λ_{ex}=330 nm
λ_{ex}=340 nm
λ_{ex}=350 nm
λ_{ex}=425 nm
λ_{ex}=540 nm
Intensity (a.u.)
Wavelength (nm)
0
100
200
300
400
500
600
300
400
500
600
700

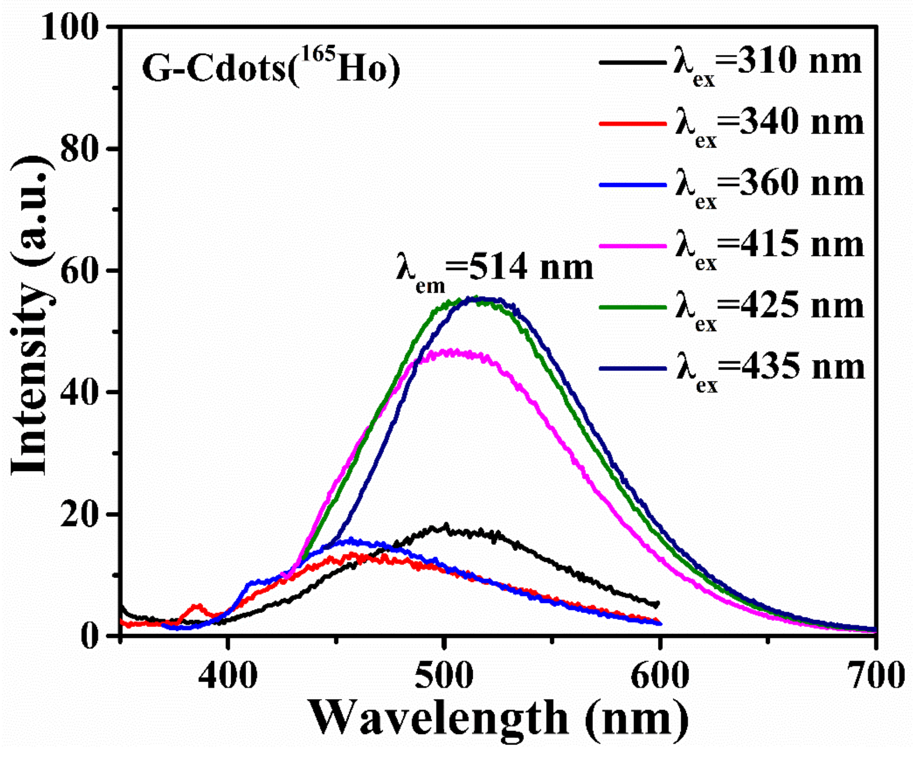
G-Cdots(^{165}Ho)
λ_{em}=514 nm
λ_{ex}=310 nm
λ_{ex}=340 nm
λ_{ex}=360 nm
λ_{ex}=415 nm
λ_{ex}=425 nm
λ_{ex}=435 nm
Intensity (a.u.)
Wavelength (nm)
0
20
40
60
80
100
400
500
600
700

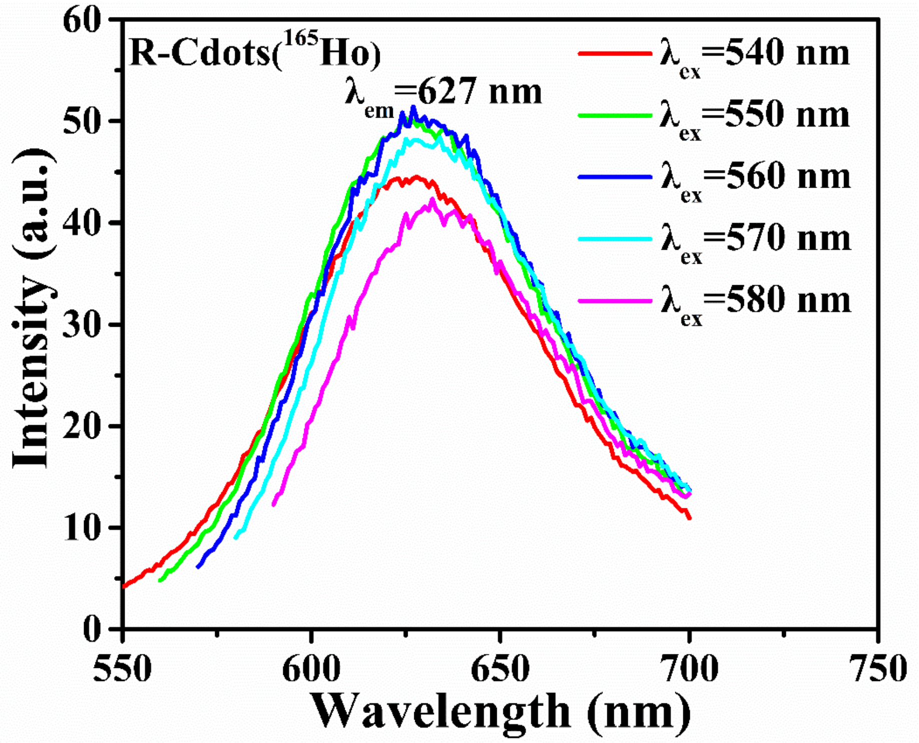

Figure S2 The photoluminescence quantum yield (QY) of MC-Cdots(^{165}Ho) was determined to be 42.6%, 8.82%, and 6.92% for B-, G-, and R-Cdots(^{165}Ho), respectively

Table S1 the Average lanthanide amount per Cdots measured by ICP-MS

**Cdots(Ln)**	***Cdots(Tb)***	***Cdots(Ho)***	***Cdots(Ce)***	***Cdots(Pr)***	***Cdots(Lu)***	***Cdots(La)***	***Cdots(Tm)***
**Ln(mass%)**	**12.79**	**19.19**	**3.72**	**2.99**	**6.47**	**2.10**	**12.20**

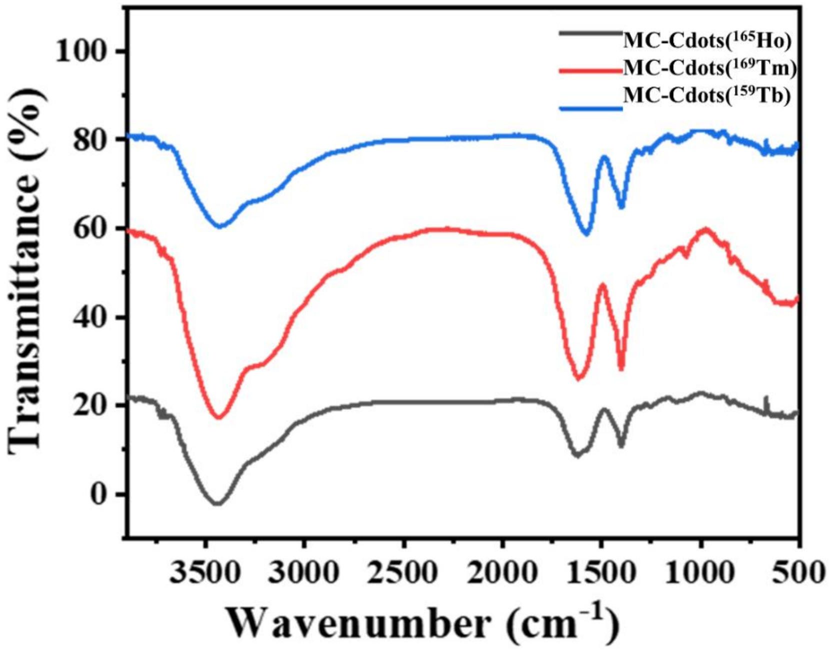

Figure S3 FT-IR spectra of MC-Cdots(^{165}Ho), MC-Cdots(^{169}Tm), and MC-Cdots(^{159}Tb), respectively.

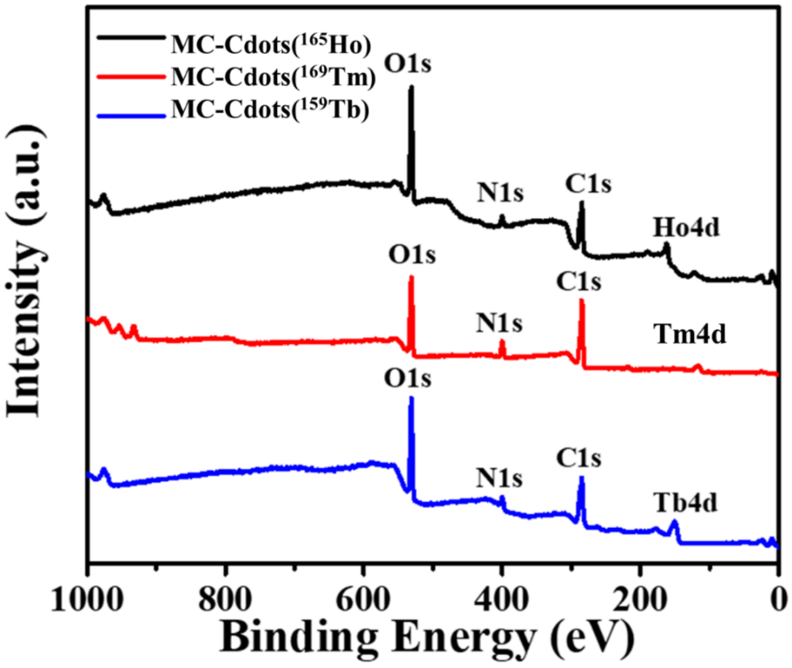

Figure S4 Full Scan of XPS spectrum of MC-Cdots(^{165}Ho), MC-Cdots(^{169}Tm), and MC-Cdots(^{159}Tb), respectively.

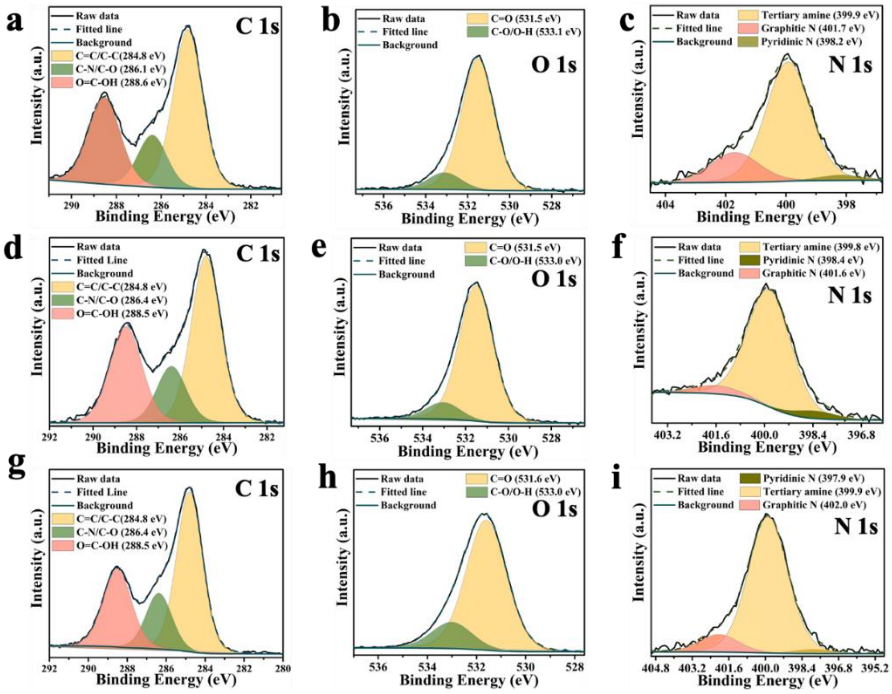

Figure S5 (a-c) High-resolution C 1s, O1s and N 1s XPS spectra of MC-Cdots(^{165}Ho). (d-f) High-resolution C 1s, O1s and N 1s XPS spectra of MC-Cdots(^{159}Tb). (g-i) High-resolution C 1s, O1s and N 1s XPS spectra of MC-Cdots(^{169}Tm).

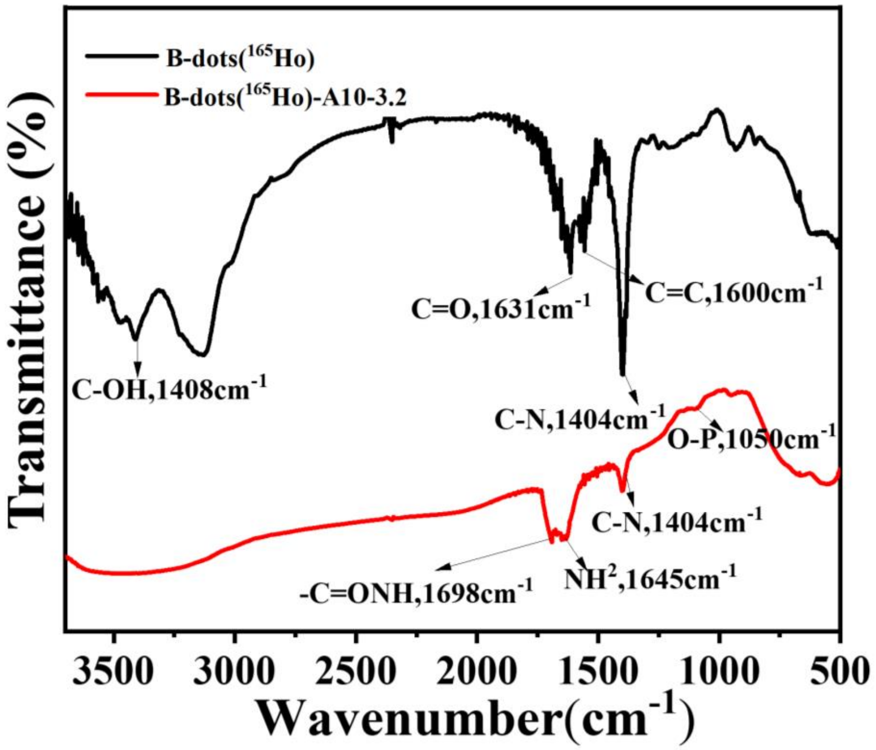

Figure S6 FT-IR spectra of B-Cdots(^{165}Ho) and B-Cdots(^{165}Ho)-A10-3.2, respectively.

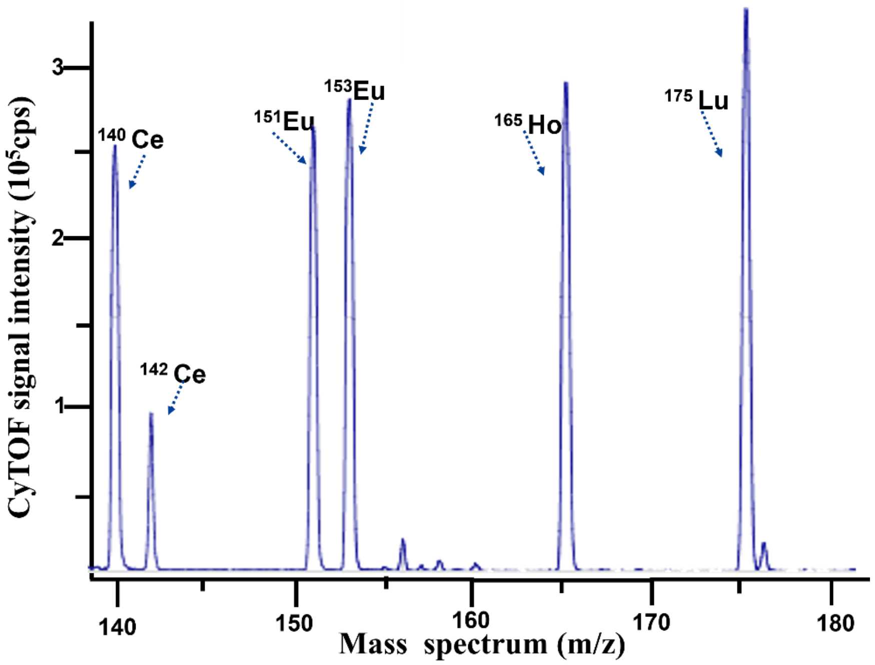

Figure S7 CyTOF mass spectra of B-Cdots(^{165}Ho)-A10-3.2 and element calibration beads (EQ beads).

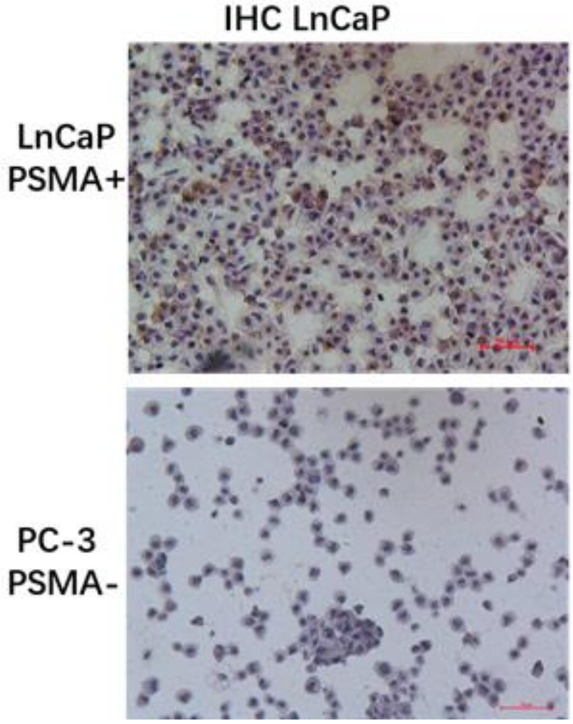

Figure S8 Traditional immunocytochemistry (ICC) of LNCaP and PC-3 staining with anti-PSMA antibody (YPSMA-1) for PSMA expression.

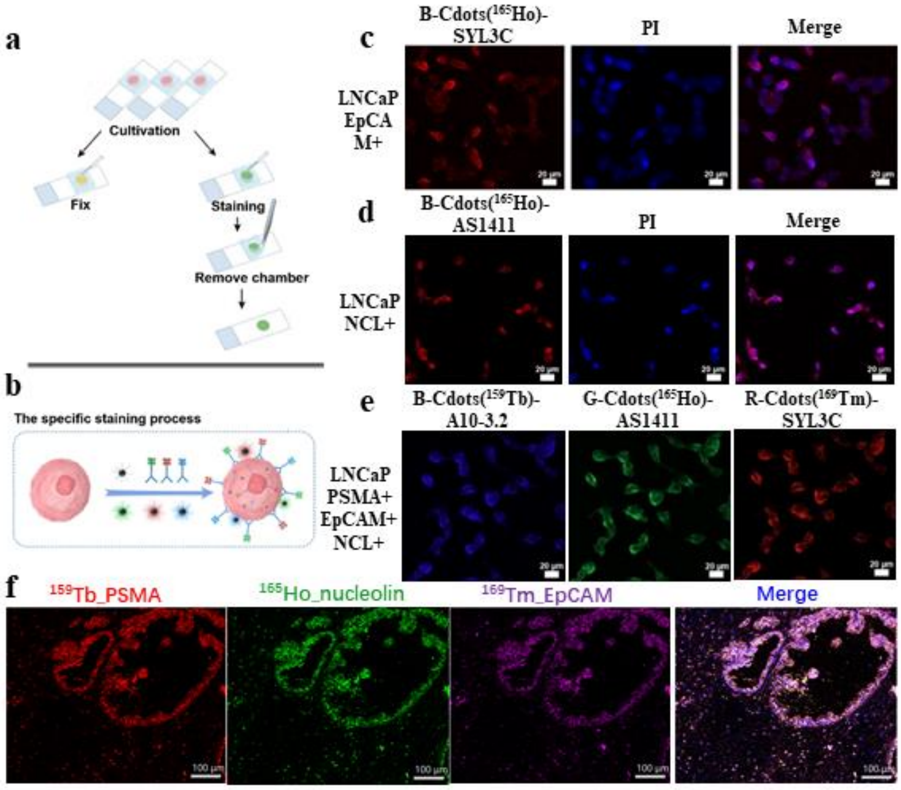

Figure S9 (a)The process of the cell lines mounted on the slides, (b) the specific staining process, (c) IFM images of the LNCaP using anti-EpCAM B-Cdots(^{165}Ho)-SYL3C aptamer, and (d) anti-NCL B-Cdots(^{165}Ho)-AS1411 aptamer. (Pseudocolor red for B-Cdots(^{165}Ho); blue for PI). (e) IFM images of the LNCaP using B-Cdots(^{159}Tb)-A10-3.2, G-Cdots(^{165}Ho)-AS1411 and R-Cdots(^{169}Tm)-SYL3C probes for co-staingin. (f) IMC images of PaC tissue sections stained by B-Cdots(^{159}Tb)-A10-3.2, G-Cdots(^{165}Ho)-AS1411 and R-Cdots(^{169}Tm)-SYL3C probes.

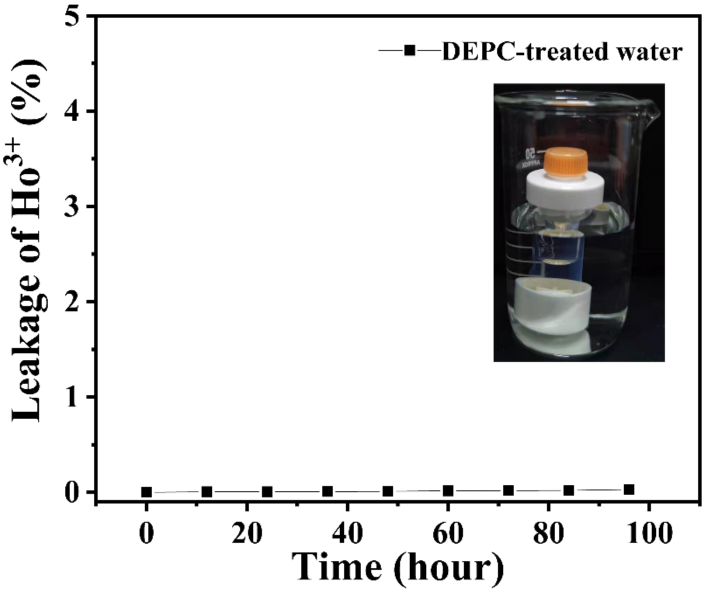

Figure S10. The leakage of Ho^{3+} of G-Cdots(^{165}Ho)-AS1411 in DEPC-treated water.

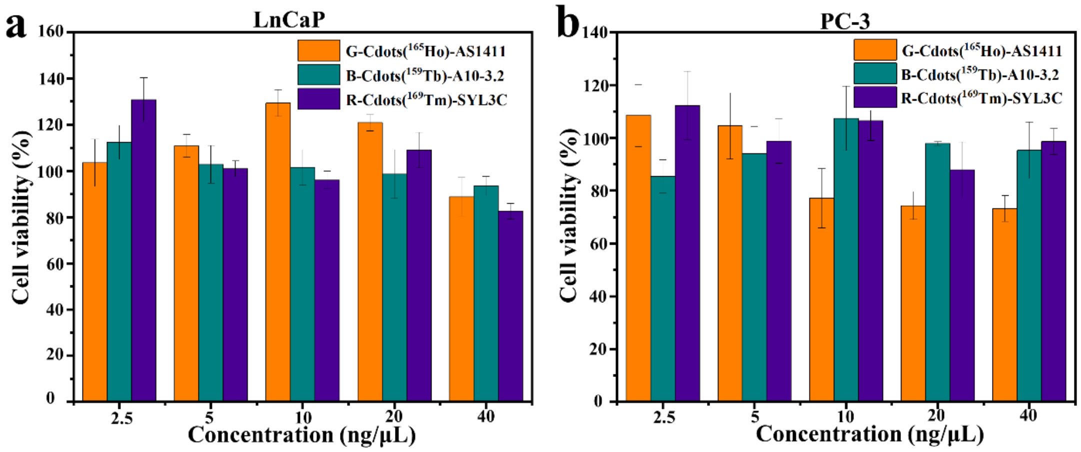

Figure S11 Cell viability of LnCaP cells (a) and PC-3 cells (b) exposed to various concentrations of B-Cdots(^{159}Tb)-A10-3.2, G-Cdots(^{165}Ho)-AS1411, and R-Cdots(^{169}Tm)-SYL3C probes (at the aptamers concentrations of 2.5, 5, 10, 20 and 40 ng/μL) for 24 h treatment. (n=3 replicates)

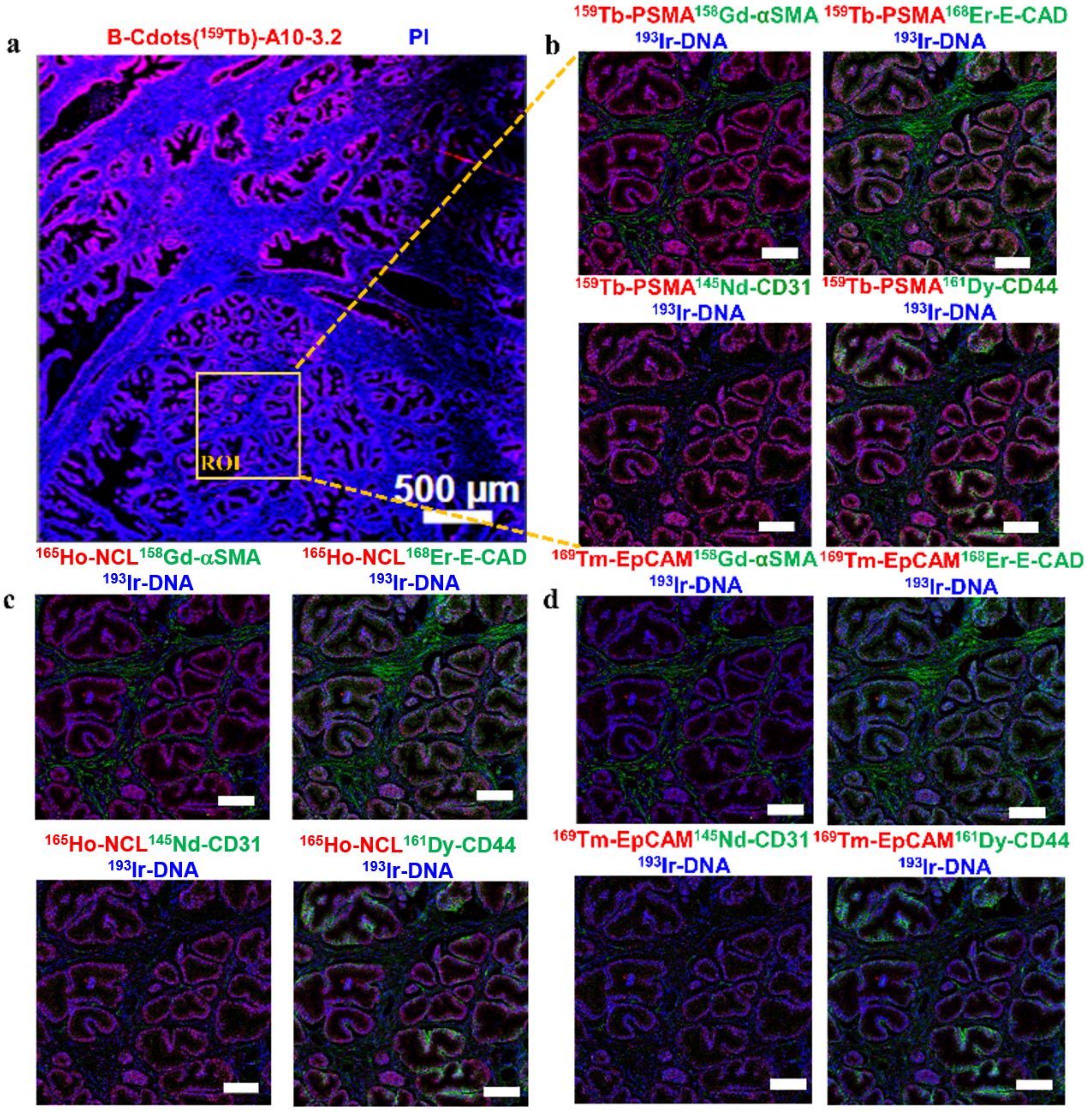

Figure S12 PaC tissue section was stained with B-Cdots(^{159}Tb)-A10-3.2, G-Cdots(^{165}Ho)-AS1411, R-Cdots(^{169}Tm)-SYL3C probes and MaxPar X8 metal-tagged antibodies analyzed by Confocal microscopy and IMC imaging. (A) ROI selected by IF. (B) Overlay of PSMA (red), α-SAM, E-CAD, CD31, CD44 (green) and DNA (Blue).

(C) Overlay of NCL (red), α-SAM, E-CAD, CD31, CD44 (green) and DNA (Blue).

(D) Overlay of EpCAM (red), α-SAM, E-CAD, CD31, CD44 (green) and DNA (Blue).

The scale bar is 100 μm.
